# Polish Society's Perspective on the Challenges Related to Childhood Cancer: Analysis of Public Opinion Research in Poland

**DOI:** 10.1002/cnr2.70297

**Published:** 2025-08-07

**Authors:** J. Pruban, K. Maleszewska, J. Antoniuk‐Majchrzak, M. Wujec, M. Czerwińska, P. Jurowczyk, Ł. Zieliński, A. Raciborska

**Affiliations:** ^1^ Department of Oncology and Oncological Surgery for Children and Youth Institute of Mother and Child Warsaw Poland; ^2^ ABR SESTA Sp. z o.o Warsaw Poland; ^3^ SYNO Poland Sp. z o.o Warsaw Poland

**Keywords:** cancer, childhood cancer, health education, pediatrics, psychooncology, social research

## Abstract

**Background:**

Treatment of children diagnosed with cancer requires coordinated, multimodal therapy that is often resource intensive and can occur over several years. In addition to physical suffering, children diagnosed with cancer experience an emotional burden that also impacts their families and communities. To best support these children and their families, we must understand society's perspective and assess its readiness to take action to improve the situation of children affected by cancer.

**Aims:**

The aim of this study was to solicit the attitudes of the Polish community towards childhood cancer. Gaining an understanding of the Polish public's awareness and knowledge regarding childhood cancer will highlight areas for future exploration and education to improve childhood cancer outcomes.

**Methods and Results:**

This study was conducted in December 2023, as a component of a collaborative research effort (Institute of Mother and Child, SYNO and ABR SESTA). The Computer—Assisted Web Interview via website research technique was used; 1002 respondents from Poland participated, maintaining the structure of Poles according to gender, age, and size of place of residence. Pediatric cancer is an important topic that concerns everyone—young people, as parents or future parents, or older people, as grandparents who often take care of children. Therefore, everyone should have elementary knowledge on this subject. This is admitted by the respondents themselves, with 65% of the total declaring that they would like to know more about childhood cancer. This is emphasized even more by parents—72%. More than half of respondents believe that the availability and quantity of materials on this topic are insufficient, and only 34% admit that in Poland there is talk about childhood cancer. Respondents agree on increasing the coverage of the topic in the media (72%).

**Conclusions:**

The perspective of Polish society, faced with the challenges related to childhood cancer, seems to be focused on the need to increase awareness, support families, access to high‐quality health care, and promote activities aimed at improving the situation of children with cancer. The analysis of the study determines the need to expand the knowledge of Polish society about childhood cancer.

## 1 Introduction

Childhood cancer remains a relatively unfamiliar topic for most of the Polish population, despite approximately 1200 new cases diagnosed annually, affecting about one in 600 children [1]. The symptoms of childhood cancer may be slightly different from those observed in adult patients [2]. Cancer in children progresses much more rapidly. Early recognition is therefore crucial, as timely diagnosis significantly improves treatment outcomes. Public awareness of childhood cancer is often limited but can be improved by better access to reliable information, strengthened health education, targeted public health campaigns, and personal experiences [3]. In Poland, numerous nongovernmental organizations (NGOs), foundations, educational institutions, and healthcare services actively work to raise awareness of childhood cancer.

Although, cancers in children are relatively rare, but when they do occur, they often follow a different course than those in adults. Many type are highly treatable if diagnosed early, making public education on symptoms and warning signs essential. For this reason, the Department of Oncology and Oncological Surgery for Children and Adolescents, Institute of Mother and Child in Warsaw, based on the survey results, has once again launched the “Show your heart” campaign. This initiative aims not only to increase awareness, but also to spark public discussion about the importance of early diagnosis and treatment of the youngest patients [4]. The aim of this study is to assess the level of awareness and knowledge of childhood cancer among the Polish population.

## 2 Methods

This study was conducted as part of a joint research project between the Institute of Mother and Child, SYNO Poland, and ABR SESTA. Ethical approval was obtained in accordance with international regulations regarding the protection of research subjects on humans (Bioethical Committee of the Institute of Mother and Child in Warsaw, opinion issued under number 4/2024 of February 23, 2024). Informed consent was obtained from all participants during the study. The survey was conducted in December 2023.

### 2.1 Research Technique

The study was designed as a cross‐sectional survey conducted using a computer‐assisted web interviewing (CAWI) method. It is a personal interview technique that uses an online interview questionnaire that you complete yourself. It followed the European Society for Opinion and Marketing Research (EOSMAR) and the Polish Association of Public Opinion and Marketing Research Firms (OFBOR) standards, and ABR SESTA holds the certificate of Quality Control of Interviewers' Work Program (PKJPA). The company carrying out the research has a panel of over 1.9 million respondents in Poland. The study sample was selected using screening questions to ensure it met the research criteria [5].

### 2.2 Study Sample

The study included 1002 respondents from Poland, selected using random sampling with quota representation to reflect the population structure in terms of gender, age, and place of residence. The final sample consisted of 52% women and 48% men, with age distribution as follows: 11% aged 18–24 years, 22% aged 25–34 years, 25% aged 35–44 years, 19% aged 45–54 years, and 23% aged 55–64 years. Quota sampling also ensured proportional representation based on place of residence, with 46% of respondents from rural areas or towns with fewer than 10 000 inhabitants, 18% from towns with 10 000–49 999 inhabitants, 17% from towns with 50 000–200 000 inhabitants, and 19% from cities with over 200 000 inhabitants. The majority of respondents had secondary education (42%) or higher education (39%), and 69% were employed at the time of the survey. Regarding financial status, 51% of respondents described their situation as “average.” Income level was not directly asked due to the high likelihood of non‐response. Instead, participants provided a subjective assessment of their financial status. Most respondents (28%) lived in households of 2–3 people. A total of 64% had children, with 32% having children aged 11–14 years and 34% having children aged 15–18 years. The largest proportion (49%) lived in small cities (≤ 1000 inhabitants).

### 2.3 Questionnaire Structure

The questionnaire consisted of 21 main questions and 9 specific follow‐up questions and was structured into three sections. The first section included screening questions, which determined whether respondents met the inclusion criteria. The second section focused on public awareness, knowledge, and attitudes regarding childhood cancer, covering aspects such as perceived prevalence, knowledge of symptoms, access to treatment, and trust in healthcare professionals. The final section collected socio‐demographic data to characterize the study sample.

The survey included single‐choice and multiple‐choice questions, Likert‐scale questions assessing opinions on cancer diagnosis and treatment, as well as filtering and control questions to verify logical consistency. Filtering logic ensured that certain questions were directed only to relevant subgroups of respondents; for example, questions about experiences with doctors were asked exclusively to parents or guardians.

### 2.4 Statistical Analysis

Statistical analyses were performed using IBM SPSS Statistics (Version 28, IBM Corp., Armonk, NY, USA). Descriptive statistics were calculated to characterize the sample. Group differences were assessed using ANOVA. When a statistically significant result was obtained for the overall test, Bonferroni post hoc tests were conducted to assess statistically significant differences between groups. The Bonferroni correction adjusts for multiple comparisons, reducing the risk of type I errors when testing for differences in categorical variables. The assumption of equal variances was verified before applying the Bonferroni test. Results were presented in tabular and graphical form, displaying frequencies and percentage distributions.

## 3 Results

### 3.1 Level of Concern Related to Lifestyle Diseases

The analysis of the results showed that lifestyle diseases are a concern for 87% of respondents (*N* = 872 out of 1002), who selected the responses “low,” “high,” or “very high.” Only a small group, about 7% of respondents declared no concern at all, with this response being more common among men, younger individuals (aged 18–24), and respondents without children in their household. Additionally, 6% (*N* = 60) of respondents selected “don't know” when asked about their level of concern regarding lifestyle diseases. A very strong level of concern was reported by one in four respondents and was found to increase with age (Table 1).

**TABLE 1:** Distribution of responses regarding level of concern.

Level of concern	*N*	%
Very high	261	26
High	431	43
Low	180	18
None	70	7
Don't know	60	6
Total	1002	100

**FIGURE 1:**
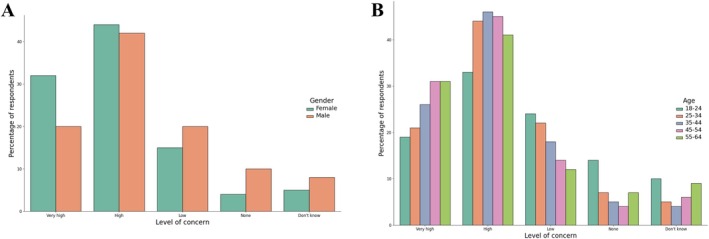
Level of concern by (A) gender (B) age group.

Difference in the level of concern were observed between women and men. Women frequently express very high and high concern, while men were more likely to declare low or no concern. This pattern is illustrated in Figure 1A. Age also played a role in determining levels of concern. Respondents in the 35–54 age groups were the most likely to report high concern, while the youngest age group (18–24) and the oldest group (55–64) showed relatively lower levels of concern. This trend is visualized in Figures 1B, where concern levels peak in middle‐aged respondents and decline among the youngest and oldest cohorts.

### 3.2 Knowledge About Childhood Cancer

#### 3.2.1 Subjective Assessment of the Level of Knowledge

**FIGURE 2:**
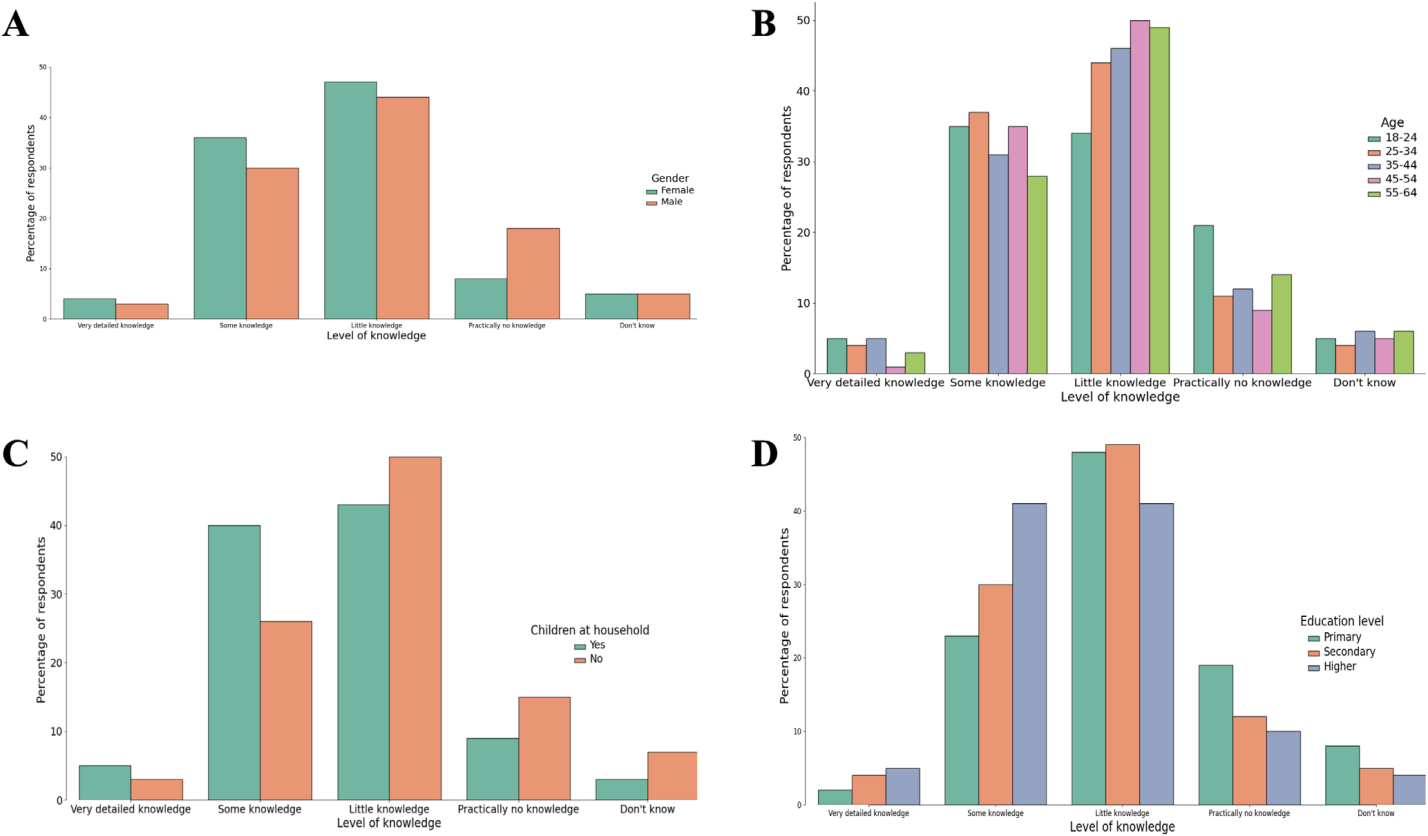
Comparison of respondents' knowledge levels about childhood cancer based on different demographic factors: (A) gender, (B) age, (C) presence of children in the household, (D) education level.

A total of 83% of respondents declared having some knowledge about childhood cancer, although in most cases (46%) this knowledge was described as low. In their subjective opinion, fewer than 1 in 20 (4%) rated their knowledge as very high, while one in three expressed confidence in their knowledge. In contrast, 13% of respondents admitted having no knowledge about childhood cancer. This group consisted more frequently of men, younger respondents (aged 18–24) compared to those aged 45–54, individuals with lower education levels, and those not living with minor children (Table 2 and Figure 2).

**TABLE 2:** Distribution of responses regarding knowledge about childhood cancer.

Level of knowledge	*N*	%
Very detailed knowledge	40	4
Some knowledge	331	33
Little knowledge	461	46
Practically no knowledge/lack of interest in the topic	130	3
Don't know	40	4
Total	1002	100

#### 3.2.2 The Scale of Cancer Incidence Among Children

More than half of the respondents (54%) believed that the number of childhood cancer cases had increased in recent years, with 14% being completely certain about this trend. This belief was more common among people who declared having knowledge of and actively following information on childhood cancer. This was also more prevalent among women and better‐educated people. In contrast, 25% of respondents believed that there had been no recent fluctuations in annual childhood cancer incidence. Epidemiological data on childhood cancer incidence are widely available and collected by public health institutions and research organizations. In Poland, the main source of such data is the National Cancer Registry, which operates under the auspices of the National Institute of Oncology—Maria Sklodowska‐Curie. Around the world, such data are collected by organizations such as the International Agency for Research on Cancer (IARC) and various national cancer registries (Table 3).

**TABLE 3:** Perceived trends in childhood cancer incidence among respondents.

Incidence of cancer among children	*N*	%
Definitely decrease	20	2
Slightly decrease	50	5
Remained at the same level	251	25
Slightly increase	401	40
Definitely increase	140	14
Don't know	140	14
Total	1002	100

#### 3.2.3 Knowledge of the Most Common Cancers in Children

**FIGURE 3:**
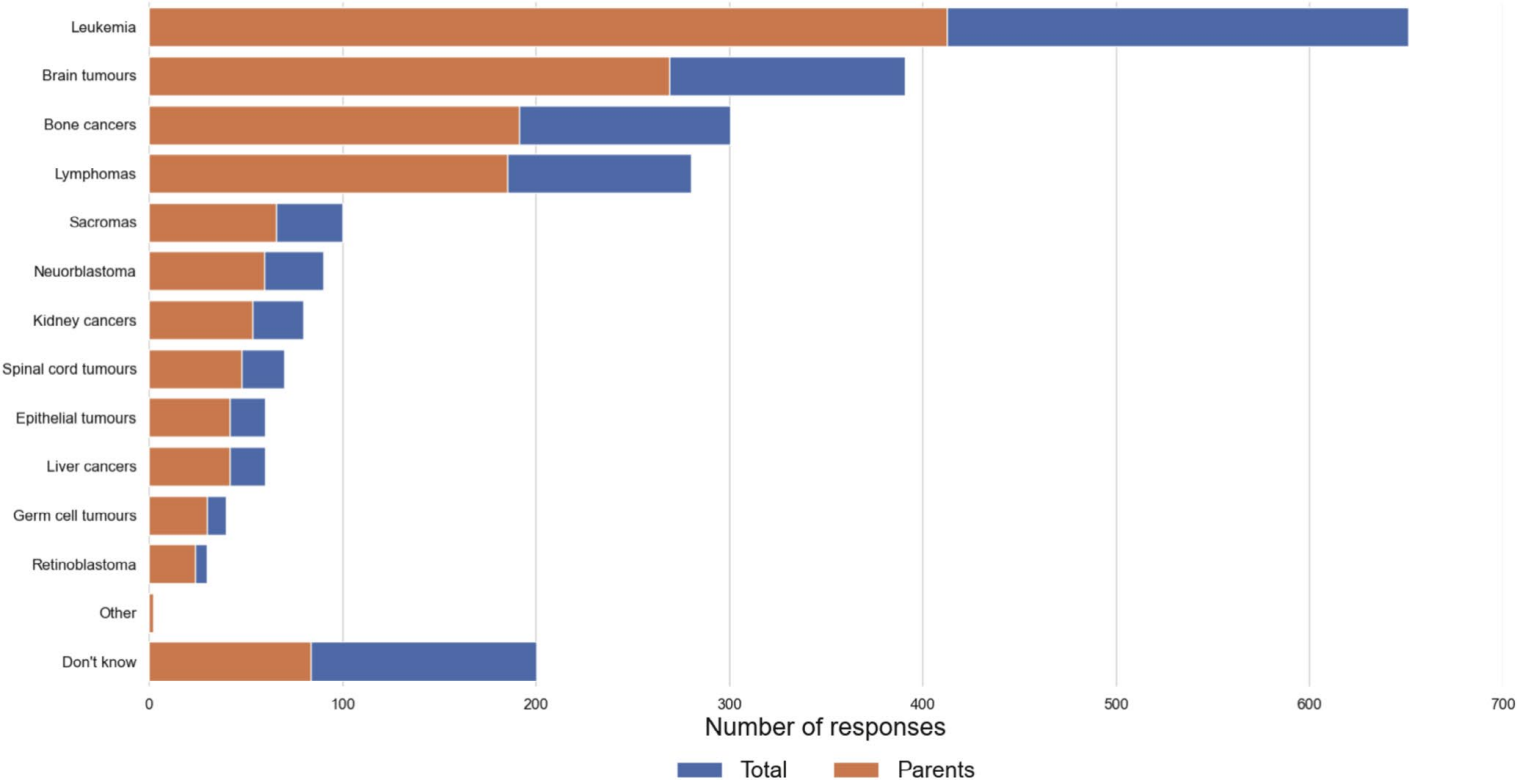
Respondents' awareness of different types of childhood cancers.

According to respondents, the most commonly recognized type of childhood cancer is leukemia (65%), followed by brain cancer (39%), bone cancer (30%) and lymphoma (28%). These results reflect respondents' perceptions of which cancers are most common in children, rather than an objective test of their awareness of all childhood cancer types. It is important to note that if a respondent did not select a specific type, it does not necessarily mean they are unaware of it—only that they may not consider it among the most common. Parents demonstrated greater awareness (33%) about childhood cancer types than people without children (22%). Additionally, parents were more likely (45%) than non‐parents to correctly identify brain tumors as one of the most common types of childhood cancer. Other cancer types listed in the survey received fewer than 10% of responses. In the scale of incidence of various types of cancers among children, neuroblastoma and spinal cord tumors hold high positions. However, they were identified by only 9% and 7% of respondents, respectively. Similarly low response rates were observed among parents, highlighting insufficient public awareness of these cancers (Figure 3).

#### 3.2.4 Knowledge of the First Symptoms of Cancer in Children

One in five respondents (20%) was unable to identify any early symptoms that could indicate the presence of childhood cancer. Among those who provided responses, the most frequently mentioned symptoms included rapid weight loss (48%), unexplained recurrent body pain (44%) and blood in urine or stool (38%).

**FIGURE 4:**
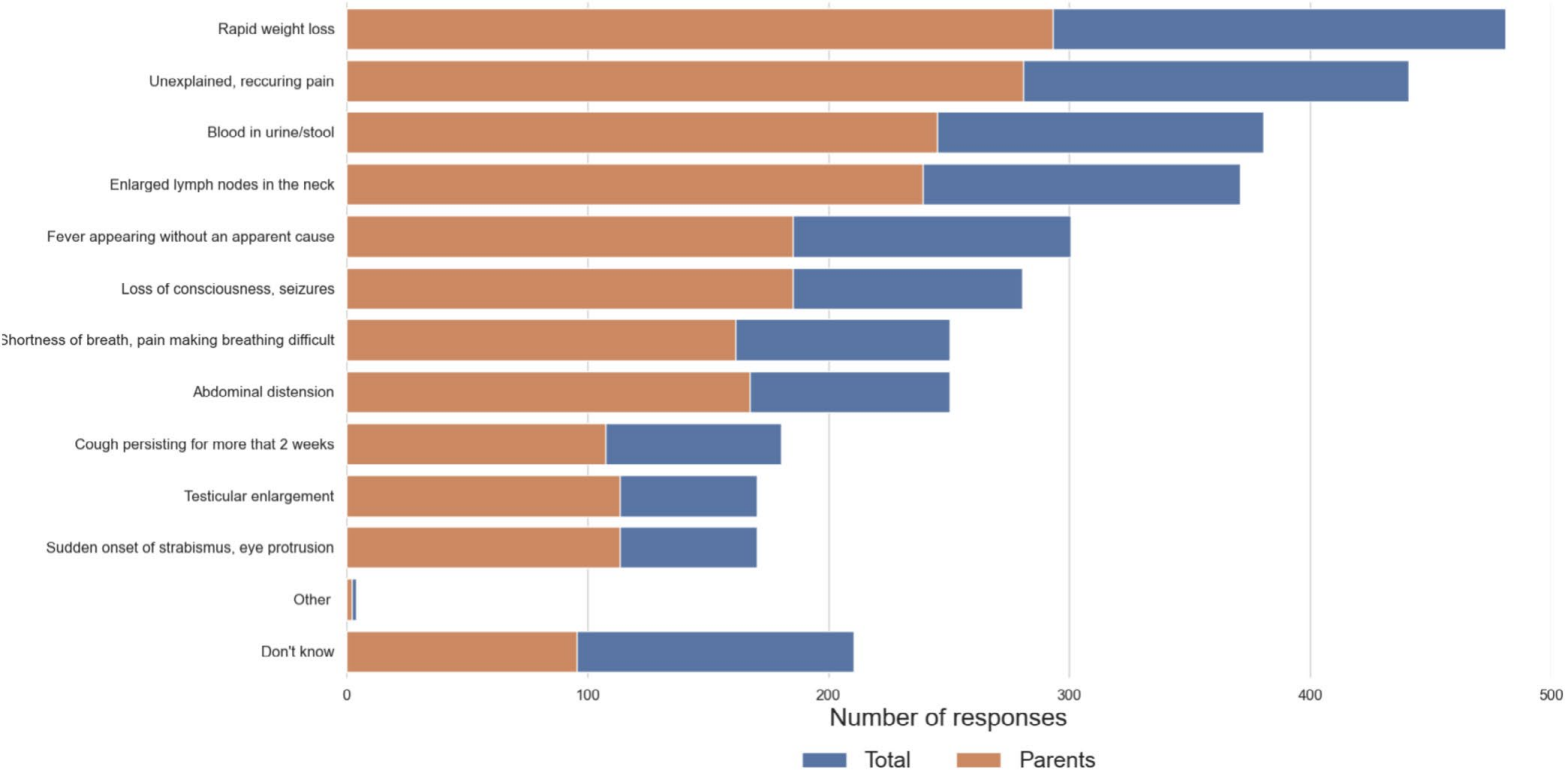
Recognition of early symptoms of childhood cancer among respondents.

On average, 3 out of 10 respondents mentioned unexplained fever and loss of consciousness as potential early symptoms, while one in four believed that shortness of breath and/or enlarged abdominal circumference could indicate cancer. Less commonly recognized symptoms included a persistent cough lasting more than 2 weeks, enlarged testicles, and sudden onset of strabismus, which were selected by 17%–18% of respondents. Parents, compared to the general sample, were less likely to struggle with identifying the first symptoms of the disease, yet 16% still answered “don't know” (Figure 4).

Recognition of alarming symptoms was particularly problematic for respondents aged 55+, as well as for those who do not live with children. Identifying symptoms was also more difficult for people with lower economic status and those who expressed little or no concern about lifestyle diseases such as cancer.

#### 3.2.5 Do Cancer and Tumour Mean the Same Thing?

**FIGURE 5:**
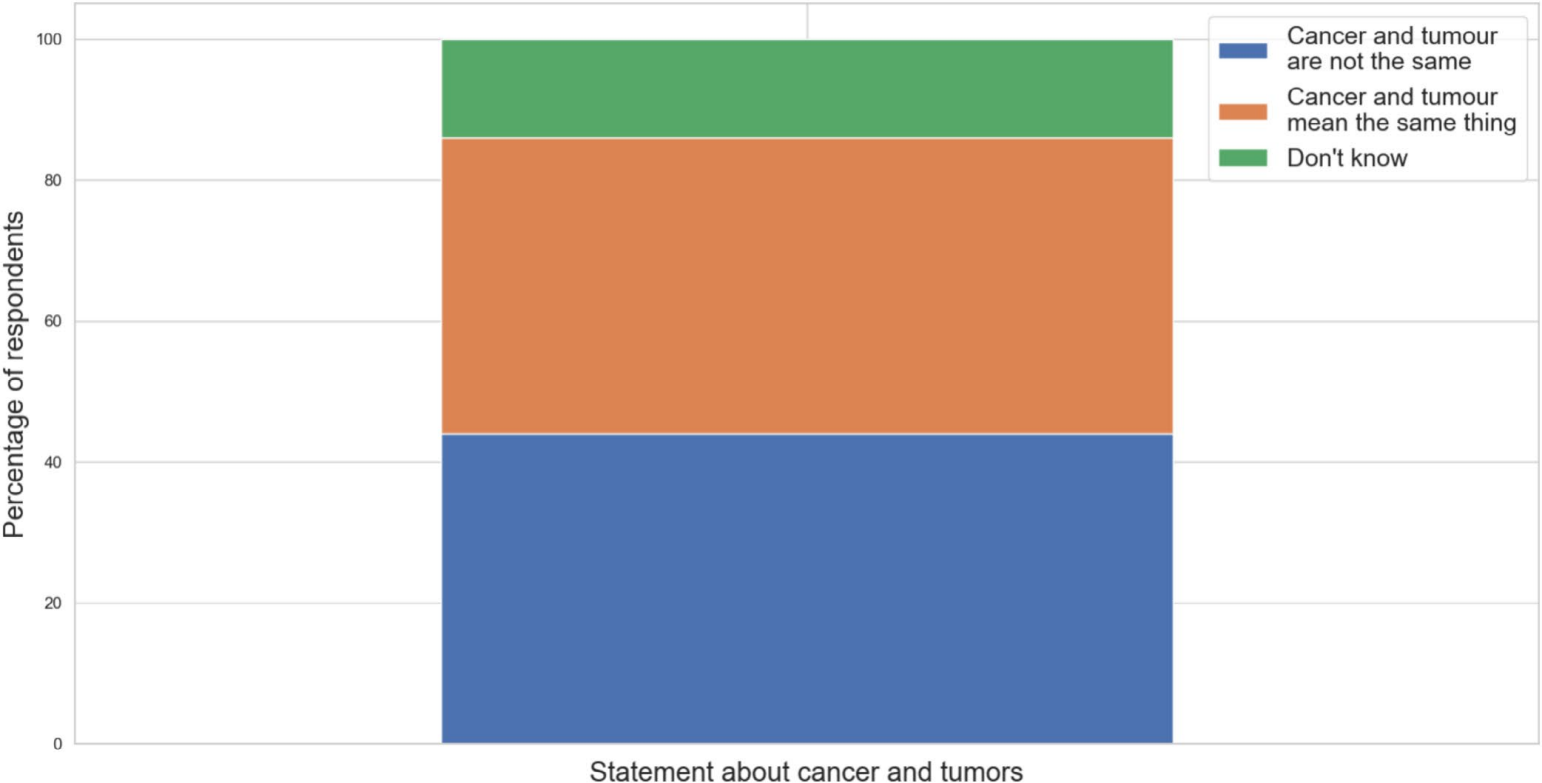
Awareness of the difference between cancer and tumors among respondents.

Nearly half of the respondents (44%) correctly distinguish between cancer and tumor. Awareness of this distinction was higher among women under 44 years of age with higher education (49%) or secondary education (42%), as well as among those living with children (47%) or more interested in the topic of childhood cancer (53%). At the same time, 42% of respondents incorrectly equated cancer with tumors, and 14% were unsure, indicating a knowledge gap in 56% of respondents (Figure 5).

#### 3.2.6 Knowledge of Modern Methods of Treating Childhood Cancer and Assessment of Their Effectiveness

**FIGURE 6:**
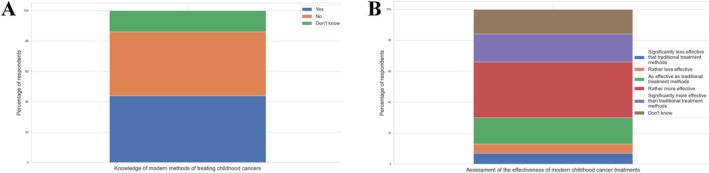
Knowledge and perceived effectiveness of modern childhood cancer treatments among respondents (A) awareness of modern treatment methods for childhood cancer, (B) assessment of the effectiveness of modern childhood cancer treatments.

A total of 46% of respondents reported having heard of modern methods for childhood cancer, including targeted therapy, clinical trials, medical robots, 3D visualizations, and artificial intelligence. Among this group, 54% believed these treatments to be more effective than traditional methods such as chemotherapy or radiotherapy. However, 36% of respondents had never encountered information about targeted therapy or immunotherapy, while some were unsure whether they had heard of them. Awareness of modern treatments was lower among men, older respondents, those without higher education, the professionally inactive, childless individuals, and those with little prior knowledge about childhood cancer (Figure 6).

#### 3.2.7 Knowledge of Modern Technologies Used in the Treatment of Cancer in Children

**FIGURE 7:**
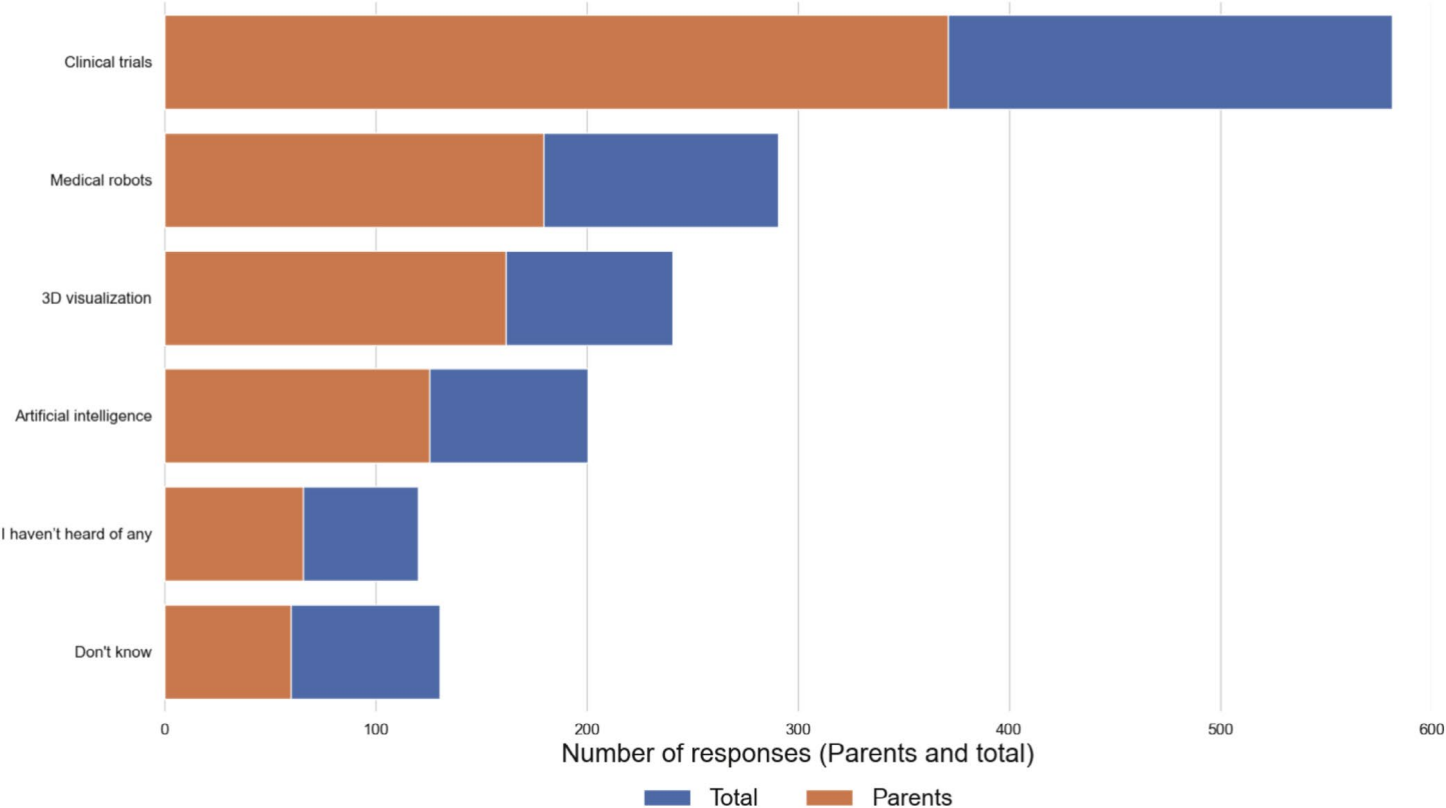
Awareness of modern childhood cancer treatment methods among respondents and parents.

One in four respondents has never heard of modern technologies used in pediatric cancer treatment (12%) or was unsure if they had encountered them (13%). Among those familiar with these technologies, the most recognized was clinical trials (58%), followed by medical robots (29%). Regarding more advanced technologies, 20% of respondents reported experience with 3D visualizations, and 25% had experience with artificial intelligence in the treatment of pediatric cancer. These methods were better known to men, highly educated individuals, working professionals, and those with greater knowledge of childhood cancer. Among parents (*N* = 598), awareness of clinical examinations (62%) and 3D visualizations (27%) was higher than in the general sample (*N* = 1002). However, even within this group, 12% of respondents had not heard of any modern technology used in childhood cancer treatment (Figure 7).

#### 3.2.8 Would Respondents Like to Know More About Childhood Cancer?

**FIGURE 8:**
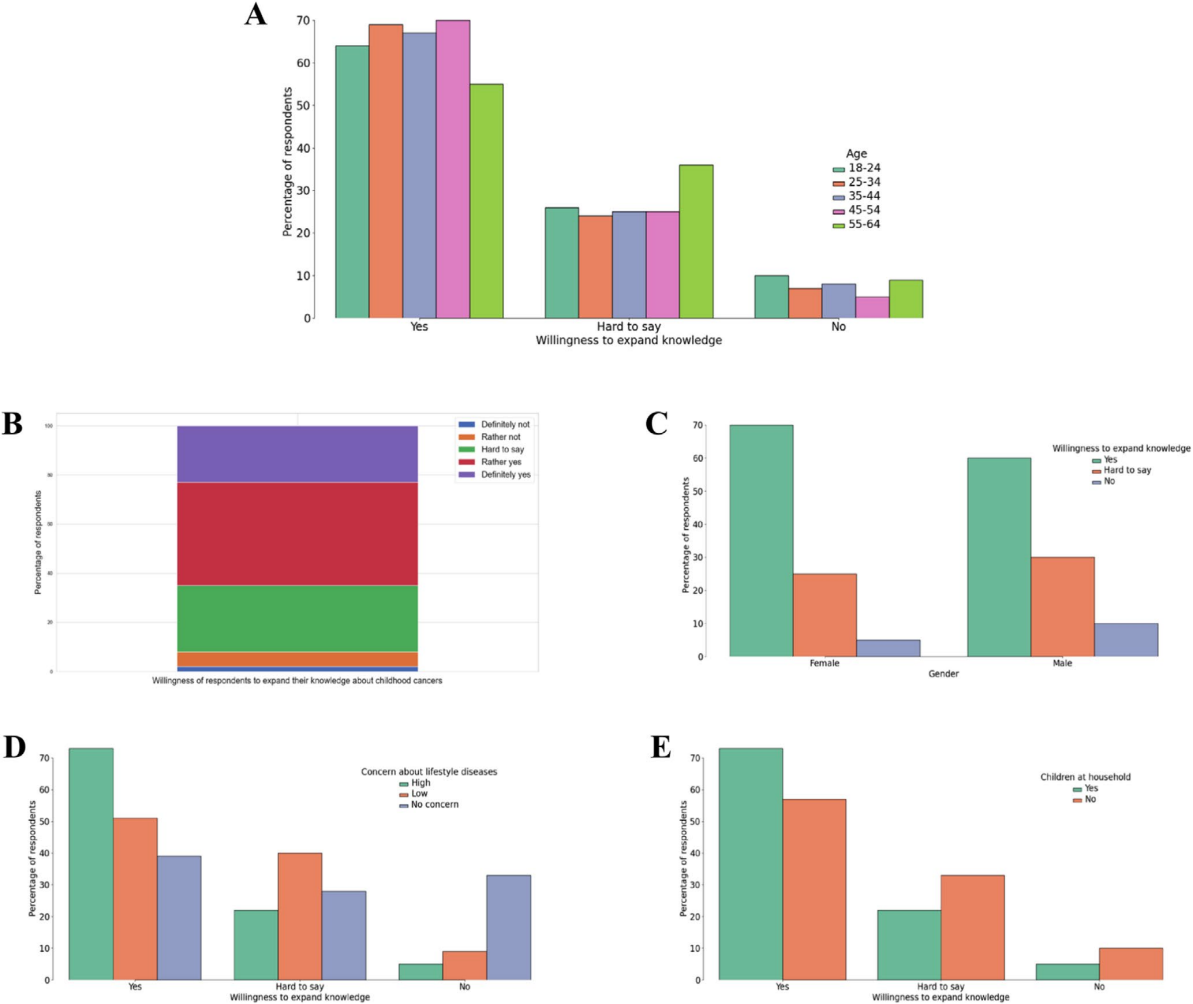
Willingness to expand knowledge about childhood cancer among respondents. (A) Interest in learning more about childhood cancer by age group. (B) Overall willingness of respondents to expand their knowledge. (C) Willingness to learn more based on gender. (D) Association between willingness to expand knowledge and concern about lifestyle diseases. (E) Willingness to expand knowledge among respondents with and without children in the household.

Many respondents (65%) expressed a desire to learn more about childhood cancer, with 23% expressing a strong interest in deepening their knowledge. Women and individuals aged 25–34 (*N* = 226) and 45–54 (*N* = 200) were more likely than older respondents and those living with children to seek further information. It is worth emphasizing that respondents who already had some prior knowledge (*N* = 370) were more likely to express interest in learning more than those who had no knowledge at all (*N* = 121). Interest was also positively correlated with fear of lifestyle diseases (*N* = 695), meaning that higher concern was associated with greater willingness to expand knowledge on childhood cancer (Figure 8).

### 3.3 Opinions on Cancer Treatment in Poland

**FIGURE 9:**
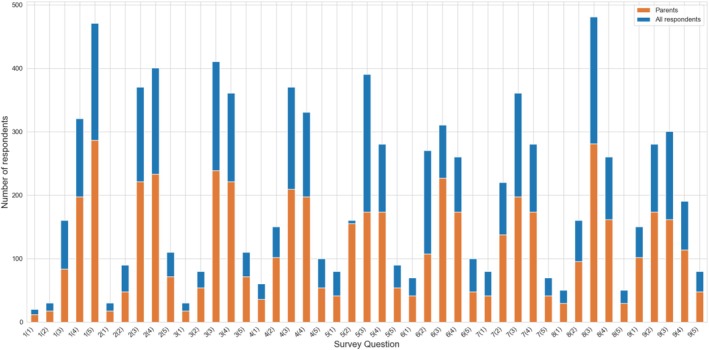
Survey responses on childhood cancer awareness and opinions on treatment. The figure presents respondents' answers to the following statements: (1) early detection of cancer in a child can significantly increase the chances of a full recovery, (2) children in Poland have the same access to modern therapies as children in other European Union countries, (3) in Poland, treatment of childhood cancers is effective, (4) many Polish oncology facilities provide access to global medical achievements and the latest therapeutic methods, (5) doctors in Poland (pediatricians/family doctors/general practitioners) thoroughly diagnose ailments in children, (6) in Poland, there are centers specializing in the treatment of specific types of childhood cancers, (7) parents have the opportunity to choose an oncology center from among all facilities throughout Poland where their children will be treated, (8) Clinical trials are also conducted in children, (9) there is a lot of talk about childhood cancer in Poland. Responses were recorded on a 5‐point Likert scale: 1—I strongly disagree, 2—I rather disagree, 3—I neither disagree nor agree/hard to say, 4—I rather agree, 5—I strongly agree.

The majority of respondents believed that early detection of childhood cancer significantly increases the chances of full recovery (79%). Additionally, 51% stated that Poland has specialized centers for treating specific types of cancer, while 47% believed that clinical trials are conducted on pediatric patients. However, opinions on other aspects of cancer treatment were more divided, with many respondents finding it difficult to make a clear assessment. Almost half of the participants (43%) believed that children in Poland do not have access to the same modern therapies that may be available to young patients in other European Union countries (Figure 9).

However, opinions on other aspects of cancer treatment were more divided, with many respondents finding it difficult to make a clear assessment.

#### 3.3.1 Do Doctors Thoroughly Diagnose Ailments in Children?

**FIGURE 10:**
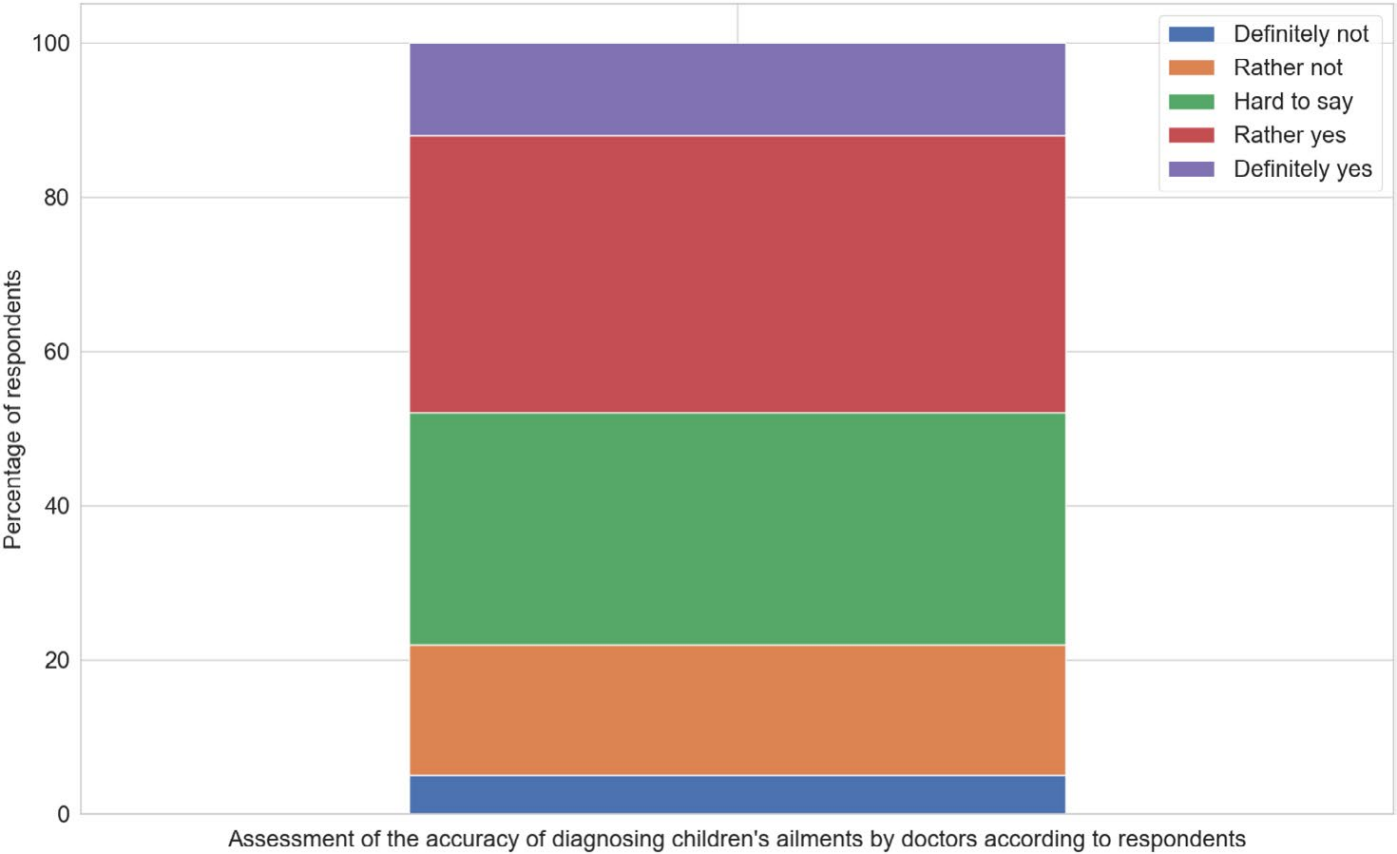
Respondents' assessment of the accuracy of diagnosing children's ailments by doctors.

More than half of the parents (52%, *N* = 598) expressed doubts about the adequacy of diagnostic procedures in pediatric care. These concerns often related to the perceived lack of clarity or confidence in the diagnostic process. At the same time, 47% of respondents emphasized that accurate diagnosis is essential for effective treatment and the long‐term health of a young patient. Regarding personal experiences, 48% believed that doctors in Poland generally take appropriate diagnostic measures, although only 12% of respondents reported that medical professionals provided as a thorough and detailed an explanation or investigation of the child's condition (Figure 10).

#### 3.3.2 Opinions About Clinical Trials

**FIGURE 11:**
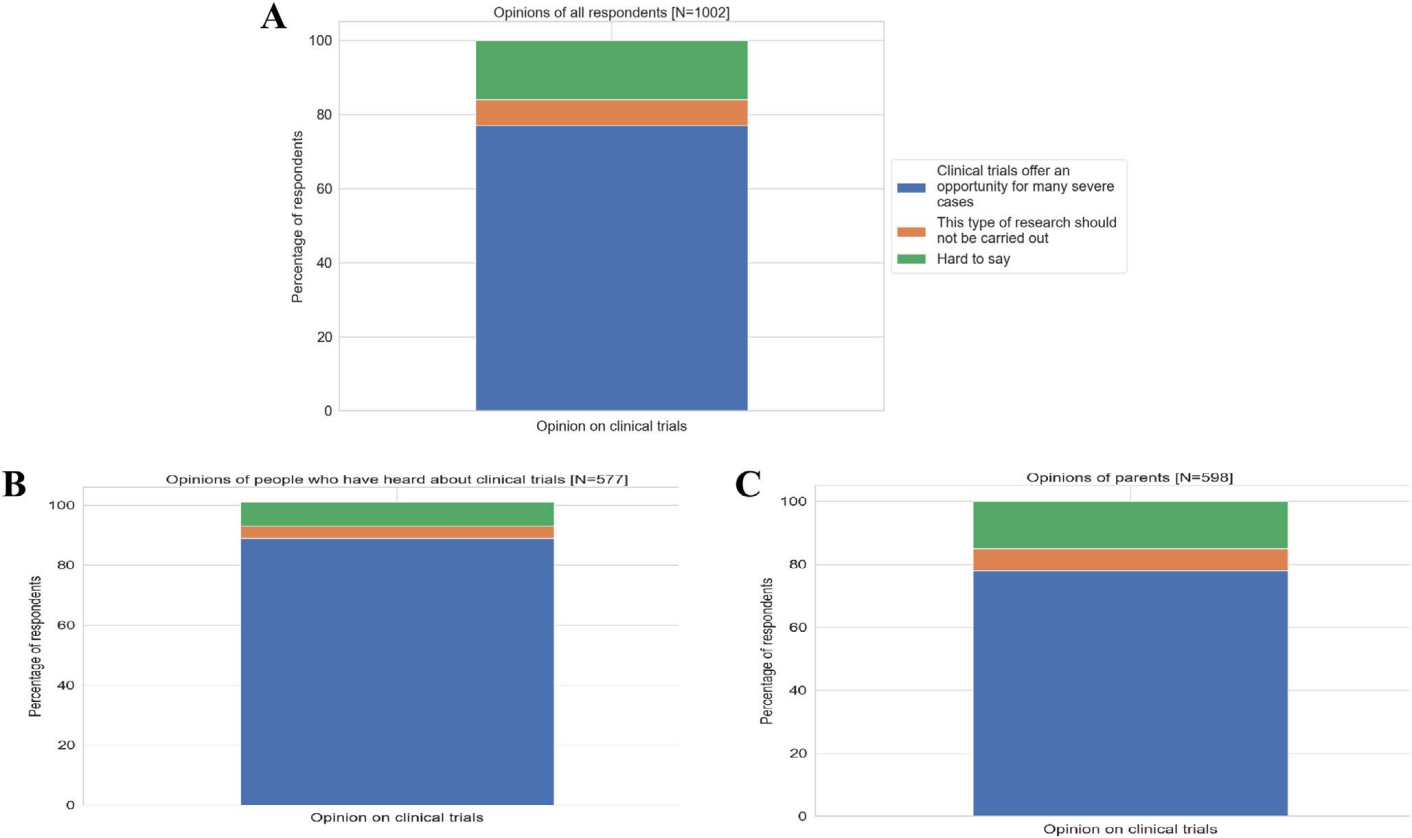
Opinions on clinical trials among different respondent groups. The figure presents the distribution of opinions on clinical trials among three groups of respondents: (A) All respondents, (B) respondents who have heard about clinical trials, (C) parents.

The vast majority of respondents (77%) viewed clinical trials as a crucial opportunity for many severe pediatric cases. Only a small group of respondents (7%) opposed conducting clinical trials on children. This view was particularly common among younger individuals (18–24 years old, *N* = 105), with the number of negative responses in this age group being half that of the total sample. In contrast, respondents aged 45–64 were significantly less likely to express opposition to clinical trials, which clearly distinguishes them from the youngest age cohort. Parents' opinions on conducting clinical trials in children closely aligned with those of the general study population (78%). However, respondents who had direct experience or knowledge of clinical trials were significantly more optimistic, with almost 90% (*N* = 577) believing that clinical trials provide a real chance to save their lives (Figure 11).

### 3.4 Presence of the Topic of Childhood Cancer in the Media and Other Sources

#### 3.4.1 Sources of Knowledge About Childhood Cancer

**FIGURE 12:**
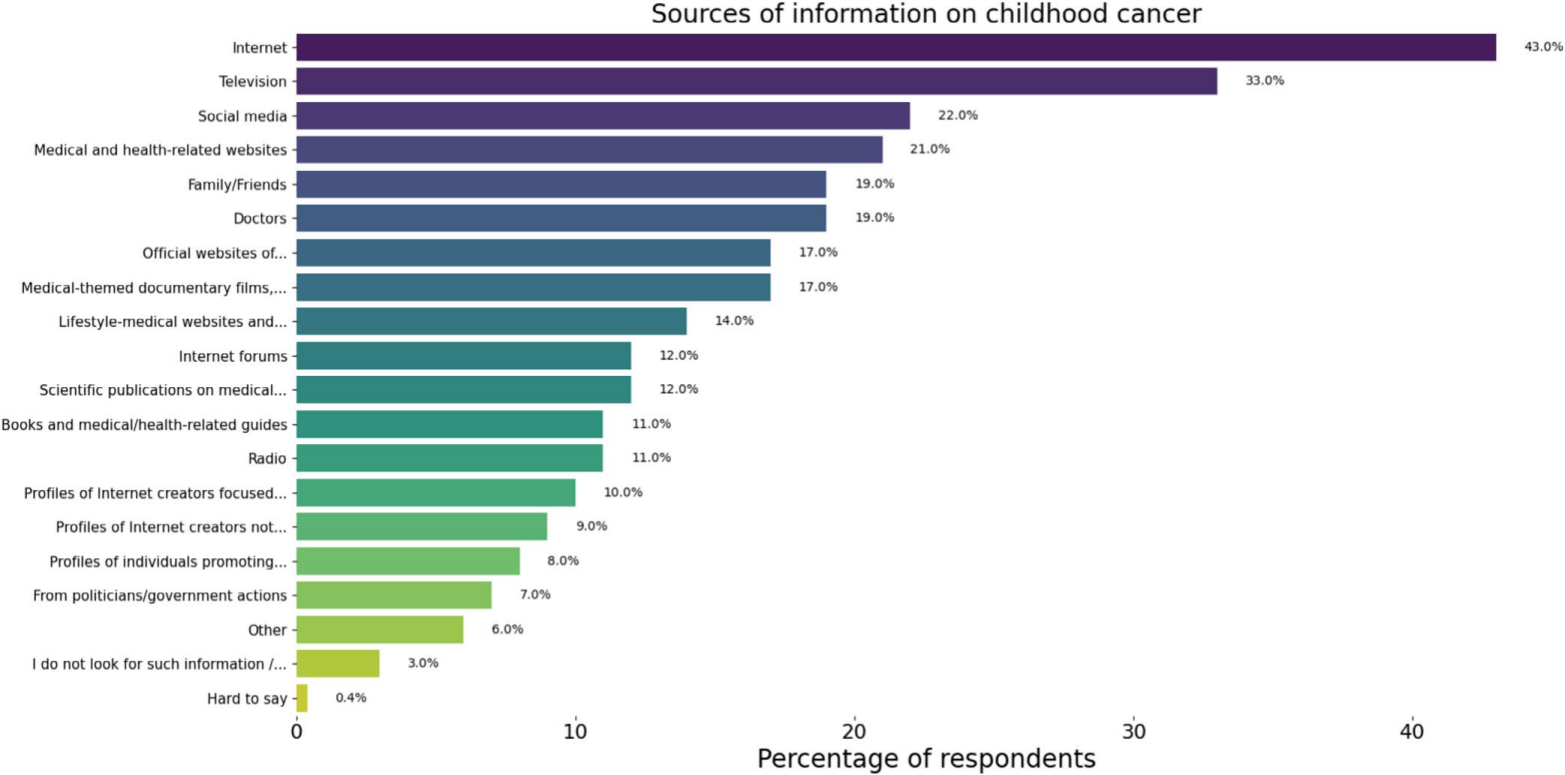
Sources of information on childhood cancer among respondents. The figure illustrates the primary sources from which respondents obtained information about childhood cancer.

The Internet is the primary source of information about childhood cancer, chosen by 43% of respondents, followed by television (33%). Approximately 20% of respondents reported seeking such information on social media, health information websites, or from family members and doctors. A smaller proportion (12%) used scientific publications on medical topics. Among those who use any information source, only 16% believe that the availability of materials on childhood cancer is sufficient, while 55% (*N* = 922) consider the available resources insufficient. Additionally, 12% of respondents reported obtaining information from the Institute of Mother and Child's website. Television was a more frequent source of knowledge for older individuals (55–64 years old—42%) and those less interested in cancer‐related topics. Women (20%) more likely than men (14%) to seek information from multiple sources, including expert opinions, and were more inclined to access strictly medical materials, i.e., scientific publications, including: Institute of Mother and Child, visited the official websites of organizations/foundations dealing with cancer and pediatric oncology, read books and guides. Women also showed greater interest in alternative medicine (9%). Respondents aged 45–54 (25%) were more likely than younger age groups (18–24: 11%; 35–44: 13%) to use lifestyle and medical websites, online forums or documentaries, and compared to the oldest age group, they more frequently sought medical opinions. According to the study, people aged 35–44 (13%) were more likely to follow social media profiles of both medical and non‐medical influencers, which distinguishes them from the oldest group (4%) in terms of interest in non‐medical content. Participants with extensive knowledge of childhood cancer were significantly more likely (16%) to obtain information from various sources, compared to 8% of those with limited knowledge. Similarly, 14% of respondents living with minors sought information from multiple sources, while only 5% of respondents without children in the household did the same (Figure 12).

**FIGURE 13:**
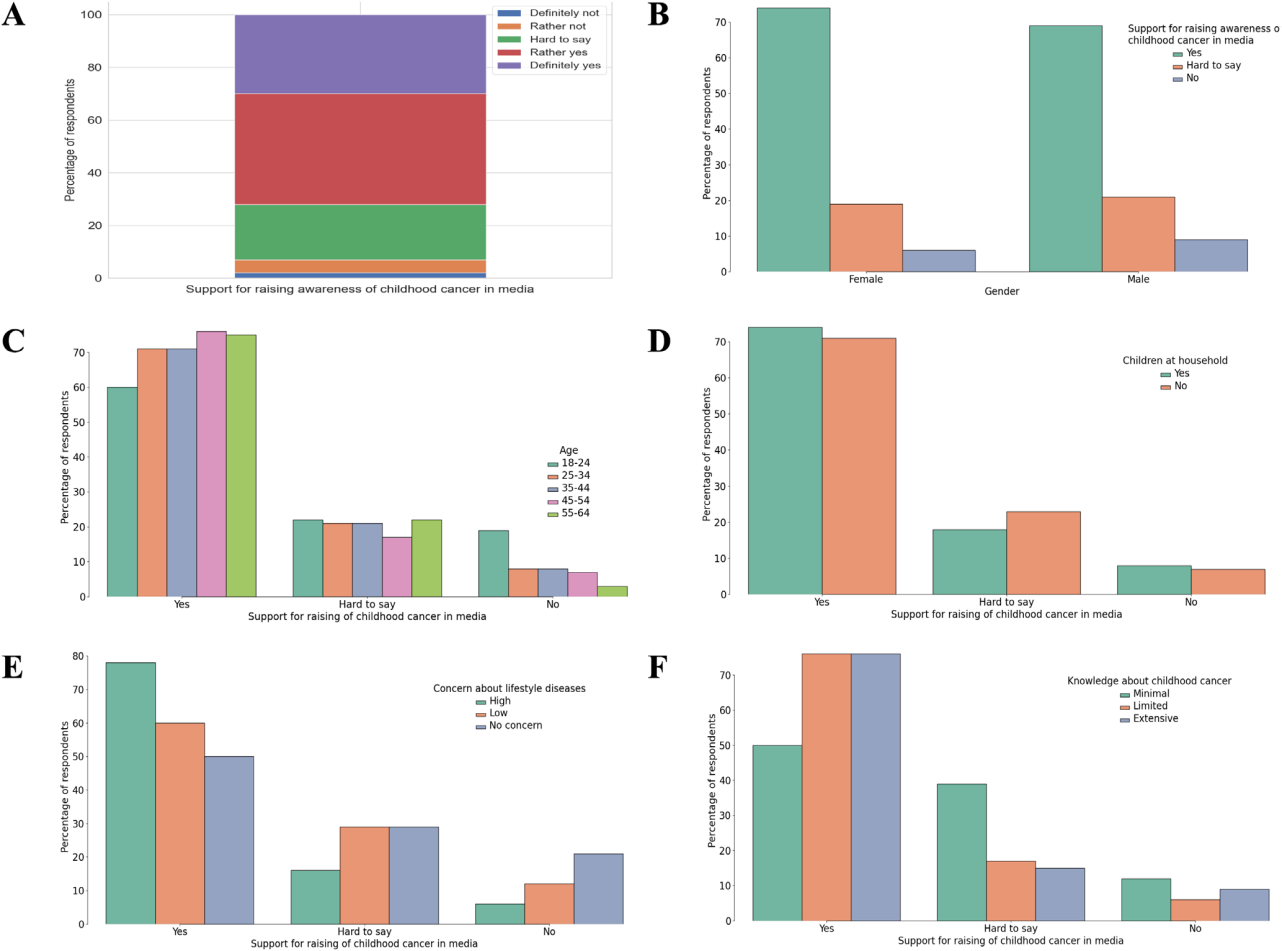
Support for raising awareness of childhood cancer in the media among respondents. (A) Overall distribution of responses regarding the importance of increasing media coverage on childhood cancer. (B) Support for raising awareness by gender. (C) Support by age group. (D) Support among respondents with and without children in the household. (E) Support based on concern about lifestyle diseases. (F) Support in relation to knowledge about childhood cancer.

The majority of respondents (72%) believed that childhood cancer should receive greater media coverage and be more frequently addressed in social campaigns. Although this opinion was not significantly influenced by socio‐demographic factors, respondents aged 45+ (76%, *N* = 200) were more likely than the youngest age cohort (60%, *N* = 105) to support greater media presence of the topic. Women (74%, *N* = 522), individuals with high levels of fear of lifestyle diseases (78%, *N* = 695), and those with children in the household (74%, *N* = 595) were also more likely to advocate for increased awareness (Figure 13).

#### 3.4.2 Health Education

**FIGURE 14:**
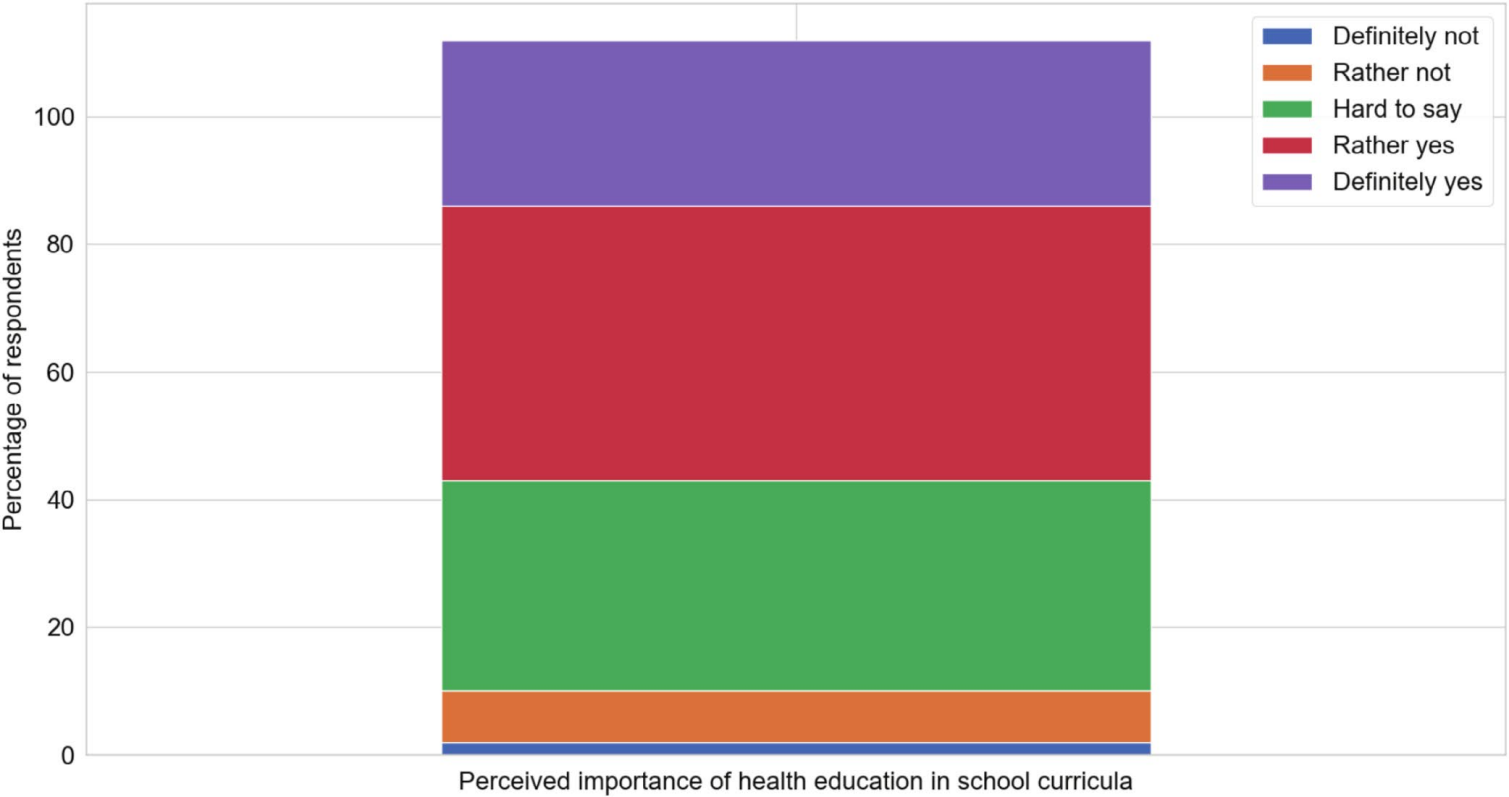
Perceived importance of health education in school curricula among respondents. The figure presents respondents' opinions on the presence of health education in school curricula.

Almost 7 out of 10 respondents (69%) supported increasing the presence of health education in school curricula while only 1 in 10 was against this idea. Individuals interested in childhood cancer were more likely (71%) to support expanding health education in schools. However, having children in the household did not significantly impact opinions on this issue. Similarly, the level of education had no major influence, though graduates of primary/vocational schools (65%) were more likely to oppose this solution compared to secondary school graduates (70%) (Figure 14).

## 4 Discussion

The study revealed a significant lack of knowledge about childhood cancer, which may lead to underestimating symptoms and delaying diagnosis. Late diagnoses are associated with a worse prognosis and often result in reduced quality of life after treatment. The findings confirm the importance of educational initiatives aimed at raising awareness of childhood cancer, particularly through social campaigns. Cancer‐related anxiety may influence how individuals engage with information about the disease. Anxiety can lead to a variety of behaviors and reactions that can influence the way an individual engages in cancer learning activities. While fear can motivate some people to seek knowledge, it may also cause others to avoid the topic entirely, as exposure to information about cancer increases distress. They may avoid reading articles, watching TV programs, or discussing cancer. The study revealed that respondents who expressed a high level of concern about lifestyle diseases were also more likely to report limited awareness of modern cancer treatment methods and clinical trials. Although no formal statistical analysis was conducted to assess this relationship, this pattern may indicate that low knowledge in these areas contributes to uncertainty and distress. This suggests a potential role for targeted education to reduce anxiety and improve public understanding. Early diagnosis of childhood cancer remains challenging [6]. Clinical symptoms vary widely depending on the tumour type, location, stage, and growth rate. The initial symptoms can be nonspecific or very discreet, often resembling common childhood illnesses [7]. This may contribute to low public awareness regarding childhood cancer and its symptoms. The results indicated that women were generally more knowledgeable about the most common types of childhood cancers [8, 9]. They were more likely than men to correctly identify leukemia, brain tumors, bone tumors, and lymphomas, as well as to recognize the incidence of spinal cord tumors and neuroblastoma [10, 11]. Respondents who had children in their households or expressed greater interest in childhood cancer were also better informed. The public opinion survey provided valuable insights into awareness levels, health education, and social attitudes towards childhood cancer in Poland. It assessed public knowledge about childhood cancer prevalence, types, and symptoms, as well as perceptions of risk factors. The findings revealed barriers to understanding and supporting children with cancer, highlighting challenges in accessing reliable information. These results suggest a need for improved communication and educational efforts in this area.

Similar public perception studies conducted in other countries provide useful context for interpreting our results. A 2019 study in the United Kingdom [12] assessed public awareness of childhood and young adult cancer symptoms and revealed that only 32% of respondents felt confident in recognizing cancer symptoms in children. The most commonly recognized symptoms were similar to adult cancer campaigns (e.g., lumps, weight loss), while developmental signs (such as delayed puberty or leukocoria) were largely unrecognized. These findings mirror our Polish results, where respondents more frequently identified general symptoms like weight loss and pain, but poorly recognized signs specific to pediatric cancers. This suggests that childhood cancer awareness campaigns globally may be overshadowed by adult‐oriented messaging.

In contrast, a study from South India [13] highlighted distinct sociocultural challenges. While 80% of participants were aware that children can develop cancer, many respondents still associated cancer with myths and stigma, such as divine punishment or infectiousness. Over 16% of respondents believed that childhood cancer is incurable, and more than half (55.6%) did not believe that a child could return to a normal life after completing treatment. While such beliefs were not prevalent in the Polish population, the Indian study underscores the importance of tailoring education to combat cultural misconceptions, which may differ substantially between high‐income and low‐to‐middle‐income countries.

### 4.1 Limitations of the Study

As with any public opinion survey, this study has certain limitations. Respondents' answers may have been based on general beliefs, personal experiences, or emotional reactions, rather than objective or verified knowledge. While the goal of the study was to assess the range of public awareness and opinions about childhood cancer, it is important to acknowledge that short surveys may not fully capture the complexity of the subject. Additionally, although the sample was selected to be representative of the Polish population by age, gender, and place of residence, it may not fully reflect other unmeasured factors, such as socioeconomic status or health literacy, which could influence perceptions and responses. It is essential to acknowledge these limitations and consider them when interpreting the study results.

### 4.2 Clinical Implication

The study revealed gaps in public recognition of certain common types of childhood cancer—particularly neuroblastoma and spinal cord tumor. Only a small percentage of respondents were aware of the incidence rates of these cancers. This limited recognition may reflect a general lack of public awareness of these conditions. As a result, early warning signs could be misattributed to less serious illnesses, potentially contributing to delays in diagnosis and treatment [7].

### 4.3 Conclusions

Official data indicate that the incidence of childhood cancer in Poland has remained stable for several years, yet most respondents held the false belief that the number of cases is increasing. The study also revealed insufficient knowledge about both standard and modern cancer therapies. While medical advancements, clinical trials, and improved diagnostic and treatment methods have been introduced in Poland over the past decades, there is still a gap between Poland and Western European countries in terms of access to cutting‐edge therapies [14]. Ignorance of modern technologies in the treatment of childhood cancer may increase anxiety among respondents. Additionally, respondents expressed uncertainty regarding whether Polish oncology facilities provide access to world‐class medical achievements and whether doctors conduct thorough diagnostic evaluations of childhood ailments. The difficulty and inconsistency in assessing these aspects highlight a need for better dissemination of medical knowledge. Based on our findings, several concrete actions can be recommended to enhance public engagement and knowledge regarding childhood cancer in Poland. First, national awareness campaigns should be implemented, focusing on early symptoms, the most common types of childhood cancer, and the importance of timely intervention. Integrating cancer‐related content into school health education curricula—tailored to different age groups—could further strengthen early recognition and prevention efforts. In addition, educational materials such as videos, infographics, and social media content should be developed and disseminated, particularly targeting younger adults and men, who were found to have lower levels of awareness. Collaboration between health institutions and NGOs should be supported to ensure consistent, evidence‐based messaging across all platforms. Furthermore, training programs for primary care physicians and educators should emphasize the recognition of warning signs and appropriate communication with families. Finally, increasing the visibility and transparency of clinical trials in pediatric oncology—including clear information on their purpose, safety measures, and potential benefits—may help to build public trust and encourage broader acceptance. Collectively, these strategies could support earlier diagnosis, reduce diagnostic delays, and promote equitable access to reliable medical information, ultimately contributing to improved outcomes for children with cancer.

From a societal perspective, the findings indicate a strong demand for increased awareness, family support, access to high‐quality health care [15] and initiatives aimed at improving the situation of children with cancer [16]. The study emphasizes the urgent need to expand public knowledge of Polish society about childhood cancer and its treatment.

## Author Contributions

**J. Pruban:** writing original draft, review and editing, conceptualization. **K. Maleszewska:** project administration, conceptualization. **J. Antoniuk‐Majchrzak:** writing review and editing, visualization. **M. Wujec:** conceptualization. **M. Czerwińska:** data curation, statistical analysis. **P. Jurowczyk:** data curation, statistical analysis. **Ł. Zieliński:** data curation, statistical analysis. **A. Raciborska:** writing review and editing, conceptualization, supervision.

## Conflicts of Interest

The authors declare no conflicts of interest.

## Data Availability

The data that support the findings of this study are available from the corresponding author upon reasonable request.
